# Artificial complementary chromatic acclimation gene expression system in *Escherichia coli*

**DOI:** 10.1186/s12934-021-01621-3

**Published:** 2021-07-05

**Authors:** Dwi Ariyanti, Kazunori Ikebukuro, Koji Sode

**Affiliations:** 1 Department of Biotechnology and Life Science, Graduate School of Engineering, Tokyo University of Agriculture and Technology, 2-24-16 Naka-cho, Koganei, Tokyo 184-8588 Japan; 2 Faculty of Biotechnology, Sumbawa University of Technology, Olat Maras, Moyo Hulu, Sumbawa, West Nusa Tenggara 84371 Indonesia; 3 Joint Department of Biomedical Engineering, The University of North Carolina at Chapel Hill and North Carolina State University, Chapel Hill, NC 27599 USA

**Keywords:** Artificial complementary chromatic acclimation, Gene expression system, CcaS/CcaR, *Escherichia coli*

## Abstract

**Background:**

The development of multiple gene expression systems, especially those based on the physical signals, such as multiple color light irradiations, is challenging. Complementary chromatic acclimation (CCA), a photoreversible process that facilitates the control of cellular expression using light of different wavelengths in cyanobacteria, is one example. In this study, an artificial CCA systems, inspired by type III CCA light-regulated gene expression, was designed by employing a single photosensor system, the CcaS/CcaR green light gene expression system derived from *Synechocystis* sp. PCC6803, combined with G-box (the regulator recognized by activated CcaR), the cognate *cpcG2* promoter, and the constitutively transcribed promoter, the P_*trcΔLacO*_ promoter.

**Results:**

One G-box was inserted upstream of the *cpcG2* promoter and a reporter gene, the *rfp* gene (green light-induced gene expression), and the other G-box was inserted between the P_*trcΔLacO*_ promoter and a reporter gene, the *bfp* gene (red light-induced gene expression). The *Escherichia coli* transformants with plasmid-encoded genes were evaluated at the transcriptional and translational levels under red or green light illumination. Under green light illumination, the transcription and translation of the *rfp* gene were observed, whereas the expression of the *bfp* gene was repressed. Under red light illumination, the transcription and translation of the *bfp* gene were observed, whereas the expression of the *rfp* gene was repressed. During the red and green light exposure cycles at every 6 h, BFP expression increased under red light exposure while RFP expression was repressed, and RFP expression increased under green light exposure while BFP expression was repressed.

**Conclusion:**

An artificial CCA system was developed to realize a multiple gene expression system, which was regulated by two colors, red and green lights, using a single photosensor system, the CcaS/CcaR system derived from *Synechocystis* sp. PCC6803, in *E. coli*. The artificial CCA system functioned repeatedly during red and green light exposure cycles. These results demonstrate the potential application of this CCA gene expression system for the production of multiple metabolites in a variety of microorganisms, such as cyanobacteria.

**Supplementary Information:**

The online version contains supplementary material available at 10.1186/s12934-021-01621-3.

## Introduction

For the development of bioprocesses using genetically modified bacteria, the physically controllable gene expression systems are recognized as an alternative to the conventional chemical induction system, considering the cost, waste, and recycling of cultivated media. Among several physical signals, light-based regulation is ideal for the regulation of cyanobacterial genes, considering the availability of various sensing systems. Therefore, optogenetics, which uses various photoreceptors to control cell behavior directly via light exposure, has recently attracted attention for synthetic biology-based bioprocess design. Cyanobacteria harbor various light-sensing systems [1–6]. Our research group is engaged in the design of microbial bioprocesses that can be controlled by the light signals, such as the CcaS/CcaR green sensing system derived from *Synechocystis* sp. PCC6803 (PCC6803) [7]. This system was successfully introduced into PCC6803 [8], the marine cyanobacterial strain *Synechococcus* sp. NKBG 15041c (NKBG 15041c), to regulate bioprocess using wavelength of marine cyanobacteria [9, 10], and non-photosynthetic bacteria *E. coli* [11]. Furthermore, CcaS was engineered [12] and successfully applied to cyanobacteria PCC6803 and NKBG 15041c [13].

**Scheme 1:**
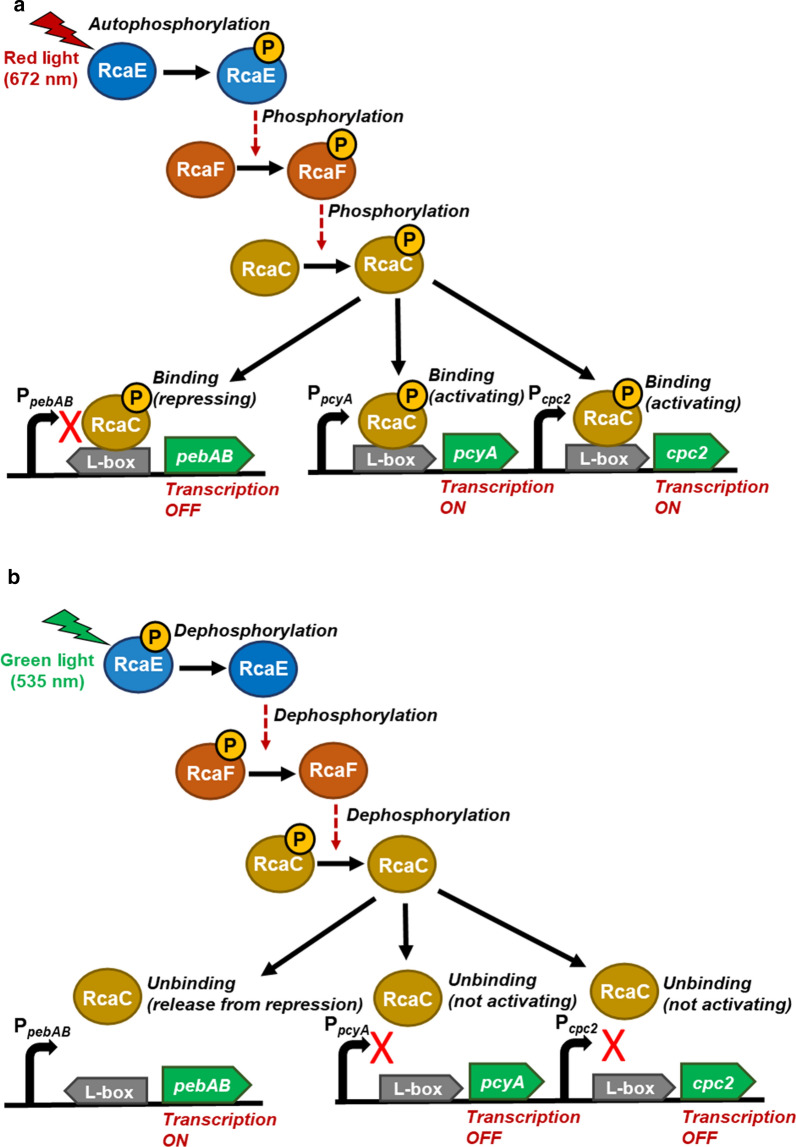
CCA system with red and green light-regulated gene expression system, derived from *Synechococcus* sp. PCC7335 [17] with some modifications. **a** Under red light, RcaE, RcaF, and RcaC are phosphorylated. Phosphorylated RcaC binding to L-box within the promoter region activates *pcyA* and *cpc2* transcription and represses *pebAB* transcription. **b** Under green light, RcaE, RcaF, and RcaC are dephosphorylated. Unphosphorylated RcaC does not bind to L-box; consequently, *pcyA* and *cpc2* transcriptions are inactivated, and *pebAB* is transcribed

One of the remarkable abilities of the cyanobacterial light-regulated gene expression system is the complementary chromatic acclimation (CCA), which enables cyanobacteria to change the protein or pigment composition of phycobilisomes and accessory light-harvesting complexes linked to photosystems during exposure to red and green light [14–16]. In CCA, cyanobacteria recognize and adapt their photosynthetic machinery to a wavelength of light by altering the phycobilisomes composition at the transcriptional level. Extensive studies have been carried out on the most representative cyanobacterial strain that displays CCA, *Fremyella diplosiphon* [14] (Additional file 1: Figure S1). The same system was also reported in the marine cyanobacterial strain *Synechococcus* sp. PCC7335 (PCC7335), which also shows type III CCA [17] (Scheme 1).

These studies have elucidated the molecular mechanism of type III CCA, which involves reversible photo-control under both red and green light. RcaE, RcaF, and RcaC protein are reportedly responsible for the regulation of type III CCA. Each protein plays a significant role in the regulation of both phycocyanin and phycoerythrin expression in a two-component regulatory system. RcaE functions as a sensor histidine kinase, and RcaF and RcaC function as cognate response regulators [4, 18–23]. Under red light, RcaE phosphorylates RcaC via RcaF, which binds to L-boxes in CCA-regulated promoters and activates the expression of red light-upregulated genes, including *cpc2*. Simultaneously, RcaC binds to L-box locates at the opposite orientation upstream of *pebAB* (green light-upregulated operon in PCC7335), at a greater distance from the transcription start site, and decreases the transcriptional activity and represses the expression of the key green light-upregulated operon, *pebAB*. Under the green light, RcaC is inactivated and cannot bind to L-boxes in CCA-regulated promoters nor activate red light-upregulated genes; simultaneously, RcaC cannot repress the expression of the key green light-upregulated operon, *pebAB*. Type III CCA recognizes and discriminates between green and red light environments using only one sensor histidine kinase, RcaE, which phosphorylates (activates) its cognate response regulator RcaC. Moreover, depending on the location of L-boxes in the promoters where activated RcaC binds, the promoter is activated or repressed [17]. The molecular mechanism of the type III CCA light-regulated gene expression regulation mechanism prompted us to design a dual light-regulated gene expression system by employing a single photosensor system to control gene expression in a recombinant microorganism.

**Scheme 2:**
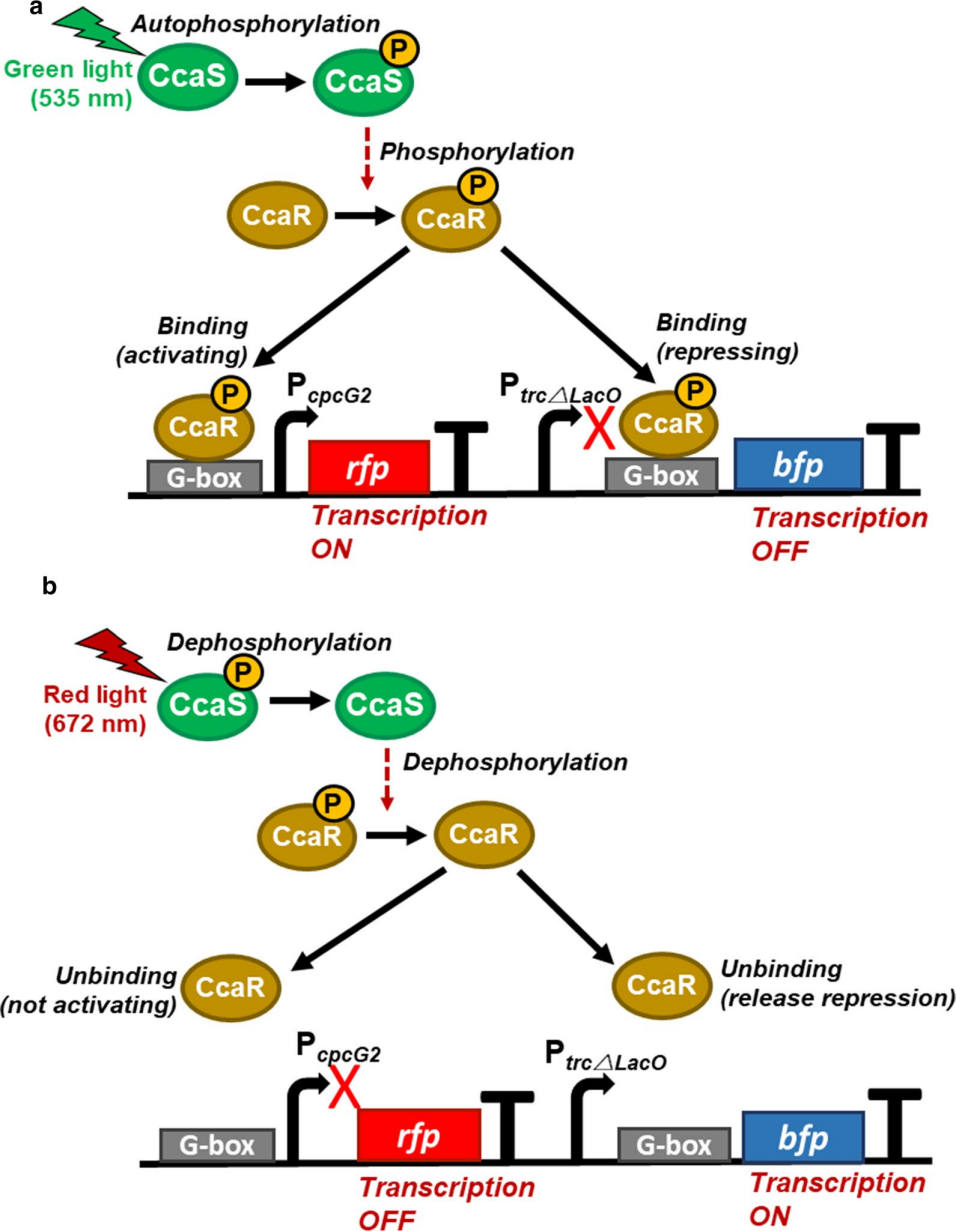
An artificial CCA system with green and red light-regulated dual gene expression system by employing a single photosensor, CcaS/CcaR, combined with G-box inserted upstream of promoters. **a** Under green light illumination, CcaS, a green light sensor, activates the response regulator CcaR. Activated CcaR binds to the G-boxes, and upregulates the *cpcG2* promoter, consequently, the *rfp * gene is transcribed. Simultaneously, CcaR binds to the G-box located between P_*trcΔLacO*_ and reporter gene, *bfp*, and downregulates the transcription of *bfp*. **b** Under red light illumination, CcaS inactivates and dephosphorylates CcaR. Inactivated CcaR is no longer able to bind to the G-boxes, and downregulates the *cpcG2* promoter; consequently, the *rfp* gene is not transcribed. Simultaneously, CcaR does not bind to the G-box located between P_*trcΔLacO*_ and reporter gene, *bfp*, transcription under the P_*trcΔLacO*_ promoter is upregulated, and the *bfp* gene is transcribed

In this study, we designed and developed an artificial CCA green and red light-regulated gene expression system, inspired by the type III CCA system in the cyanobacteria, using a single two-component regulated photosensor system, CcaS/CcaR, combined with a G-box inserted downstream of the promoters to mimic of type III CCA light-regulated gene expression (Scheme 2). We employed the CcaS/CcaR green light system derived from PCC6803 as the only photosensor. The *rfp* gene was inserted downstream, whereas the inherent CcaR binding site, G-box, was located upstream, of the *cpcG2* promoter. The *bfp* gene was inserted downstream of the P_*trcΔLacO*_ and another G-box was inserted between P_*trcΔLacO*_ and *bfp*. Under the green light illumination (Scheme 2a), CcaS activates CcaR to bind to the G-box upstream of the *cpcG2* promoter, thereby upregulating the transcription of *rfp*; activated CcaR is also able to bind to the G-box located between P_*trcΔLacO*_ and *bfp*, thereby downregulating the transcription of *bfp*. Under the red light illumination (Scheme 2b), CcaS inactivates and dephosphorylates CcaR, thereby releasing CcaR from G-boxes. Consequently, the transcription of *rfp* is downregulated, whereas the transcription of the *bfp* gene is upregulated.

To realize the designed artificial CCA gene expression system, a plasmid vector was constructed encoding the gene components of artificial CCA. *E. coli* DH5α was used as the model microorganism, after transformation with the plasmid encoding the phycocyanobilin (PCB) synthesis gene cassette, to demonstrate the expression of red fluorescent protein and repression of blue fluorescent protein under green light illumination, and the repression of red fluorescent protein and expression of blue fluorescent protein under red light illumination, which were regulated at the transcriptional level.

## Results

### Construction of red light-regulated gene expression system using a green light sensor

First, we constructed a plasmid for the red light-regulated gene expression system, by mimicking the Rca system of the CCA system of PCC7335, but utilizing the CcaS/CcaR green light-sensing system of PCC6803 as the photosensor. The CcaS photoreceptor and cognate response regulator CcaR together with their native promoters were assembled with a strong P_*trcΔLacO*_ promoter, which was modified by inserting a G-box sequence, between P_*trcΔLacO*_ and the structural gene of a *bfp* as a reporter gene (BBa_K592024) [24]. The final construct, named pBR-RSS-*bfp*, functioned as a red light-regulated plasmid.

**Fig. 1:**
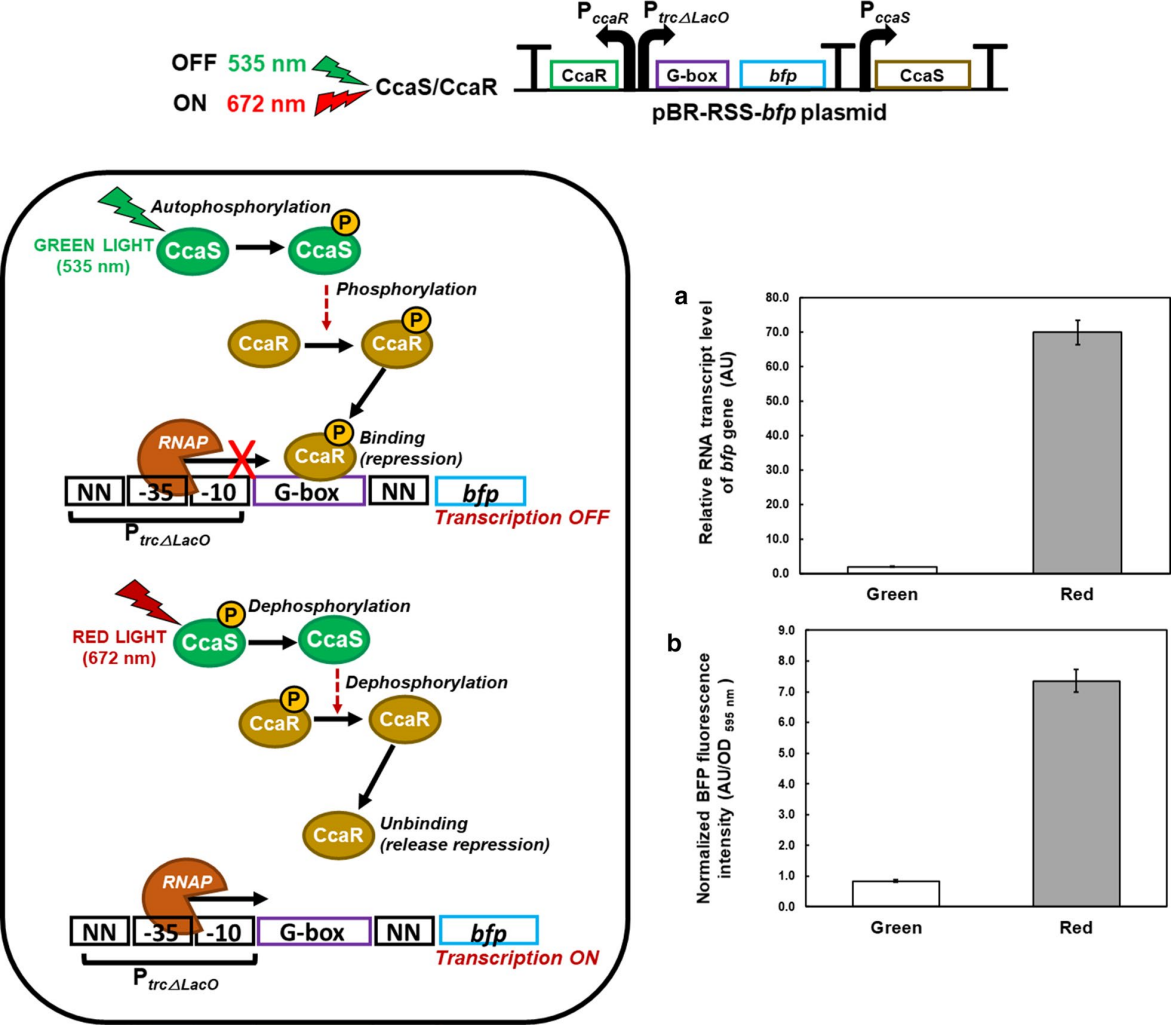
Evaluation of *E. coli* transformants harboring pBR-RSS-*bfp* after 15 h under red or green light. **a** Transcriptional analyses of the *bfp* gene normalized by 16S *rRNA* housekeeping gene. **b** Translational analysis using fluorescence assay; BFP is normalized by cell density at OD 595 nm. Data represent mean  ±  SD from three independent experiments

After 15 h of red light illumination, the transcription of the *bfp* reporter gene derived from the pBR-RSS-*bfp* plasmid reached its highest level of approximately 69.9 AU; this was 36.2-fold higher than that achieved under green light illumination (1.93 AU; Fig. 1a). To represent BFP at the translational, a fluorescence intensity assay was used. The fluorescence intensity of BFP was 7.35 AU/OD _595 nm_ after red light illumination; this was 8.75-fold higher than that after to green light illumination (0.84 AU/OD _595 nm_). These results indicated that the constructed red light-regulated gene expression system functioned, and that gene expression was induced under red light but repressed under green light.

### Construction and characterization of artificial CCA gene expression system

The artificial CCA gene expression system was constructed by inserting a red light sensor region (P_*trcΔLacO*_-G box-*bfp*-Terminator), from the pBR-RSS-*bfp* plasmid, downstream of the *rfp* gene, from the previously constructed pBR-GSS-*rfp* plasmid [11], under the regulation, a single photosensor derived from two-component regulated photosensory system CcaS/CcaR green light-sensing system of PCC6803. The response of pBR-GSS-RSS plasmid, to red and green light illumination was evaluated, at the transcriptional and translational levels under pseudo-continuous cultivation [25].

After 18 h of cultivation at 30 °C, in an incubator shaker at 140 rpm, under dark light, the cells transformants was reached the exponential phase. Decreasing the temperature to 25 °C, and with media replacement every 12 h, the stationary phase was maintained until 54 h of the cultivation, at which point the transformants were exposed to the designated light illuminations.

**Fig. 2:**
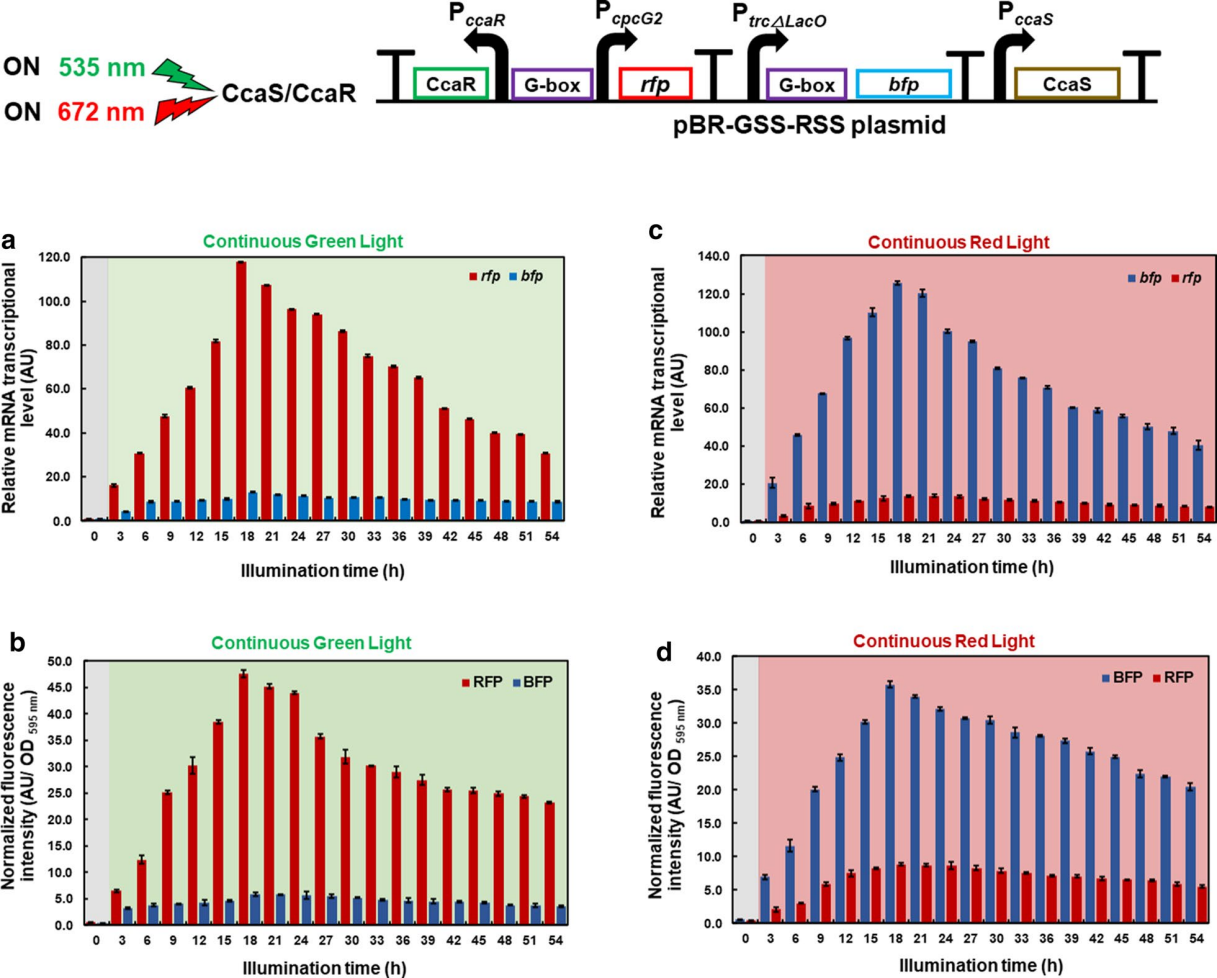
Transcriptional and translational analyses of each designated reporter gene from *E. coli* transformants harboring pBR-GSS-RSS. Under continuous green light illumination, **a** RNA transcript level of *rfp* and *bfp* reporter genes and **b** fluorescence analysis of RFP and BFP. Under continuous red light illumination, **c** RNA transcript level of *rfp* and *bfp* reporter genes and d: fluorescence analysis of RFP and BFP. The RNA transcript level was normalized by the 16S *rRNA* housekeeping gene and the fluorescence intensity was normalized by a cell density at OD 595 nm. Data represent mean  ±  SD from three independent experiments

The transcription of the *rfp* gene reached the highest level of 115.96 AU after 18 h of continuous green light illumination, which was 8.40-fold higher than that of the *bfp* gene (13.80 AU), (Fig. 2a). Additionally, the trend of RFP and BFP fluorescence intensity levels were consistent with these results, as RFP fluorescence intensity reached its highest at value 47.55 AU/OD _595 nm_ after 18 h of green light illumination, which was 8.05-fold higher than that of BFP fluorescence intensity (5.90 AU/OD _595 nm_; Fig. 2b).

In contrast, the transcription of the *bfp* reporter gene reached its highest level of 126 AU after 18 h of continuous red light illumination, which was 9.09-fold higher than that of the *rfp* gene (13.80 AU; Fig. 2c). Additionally, the BFP fluorescence intensity reached its highest value of 35.8 AU/OD _595 nm_ after 18 h of red illumination light induction, which was 4.06-fold higher than that of RFP fluorescence intensity (8.80 AU/OD _595 nm_; Fig. 2d).

These results demonstrate that the designed and constructed artificial CCA gene expression system can regulate the expression of two genes under the red and green light using a single light sensor. Here, *bfp* gene expression was induced under red light while *rfp* gene expression was repressed, whereas *rfp* gene expression was induced under green light while *bfp* gene expression was repressed, as we expected.

### Investigation of CCA under repeated red and green light illumination

**Fig. 3:**
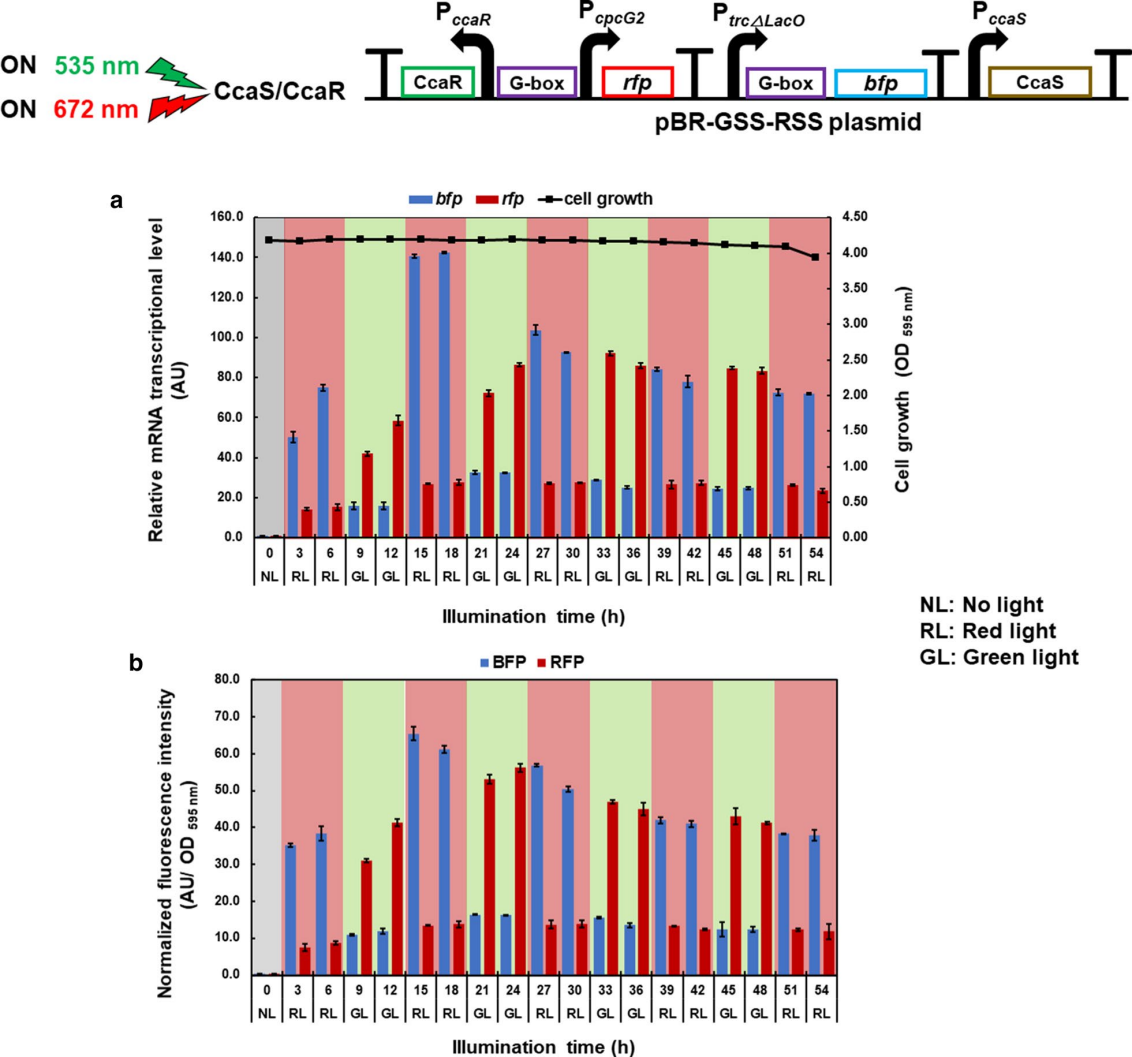
Transcriptional and translational analyses of BFP and RFP from *E. coli* transformants harboring pBR-GSS-RSS under repeated red and green light illumination under pseudo-continuous cultivation conditions. **a** *bfp* and *rfp* RNA transcription level normalized by 16S *rRNA* housekeeping gene under repeated red and green light illumination. **b** Fluorescence intensity of BFP and RFP normalized by a cell density at OD 595 nm under repeated red and green light illumination. Data represent mean  ±  SD from three independent experiments

Using pseudo-continuous cultivation conditions, the effect of the red and green light exposure cycles on the artificial CCA gene expression system were investigated. After 18 h of cultivation at 30 °C, in an incubator shaker at 140 rpm, under dark light and transformants reached the exponential phase, red light was applied and its effects at the transcriptional and translational levels were evaluated under pseudo-continuous cultivation conditions. The transcription of *bfp* reached level of 75.1 AU, after 6 h of red light illumination, which was 4.93-fold higher than that of the *rfp* gene (15.2 AU). When the light was changed to green light 6 h later, the level of *bfp* transcription decreased to 15.8 AU; in contrast, the level of the *rfp* transcription increased to 58.6 AU, or 3.69-fold higher than that of *bfp*. When the light was changed back to red 6 h later, the transcript level of *bfp* increased to 142 AU, and that of *rfp* decreased to 27.5 AU. This pattern was repeated until 54 h of illumination time (Fig. 3a).

The trend was consistent at the translational level. After 6 h of red light illumination, the BFP fluorescence intensity reached 38.4 AU/OD _595 nm_, which was 4.46-fold higher than the RFP fluorescence intensity (8.61 AU/OD _595 nm_). When changed to green light 6 h later, the fluorescence intensity of BFP decreased to 11.9 AU/OD _595 nm_ and that of RFP increased to 41.3 AU/OD _595 nm_. Moreover, when changed back to red light 6 h later, the BFP fluorescence intensity increased to 61.2 AU/OD _595 nm_ and RFP fluorescence intensity decreased to 13.7 AU/OD _595 nm._ Overall, during the red and green light exposure cycles, the expression of BFP increased under red light exposure while that of RFP was repressed, and that of RFP increased under green light exposure while that of BFP was repressed, respectively (Fig. 3b).

## Discussion

In this study, an artificial CCA gene expression system was developed using the CcaS/CcaR green light two-component regulation system derived from PCC6803. This was inspired by the single photosensor-based regulation of type III CCA reported in PCC7335, which is similar to that reported in *F*. *diplosiphon* [17], to achieve dual control of the light-regulated gene expression system in *E. coli*.

The constructed plasmid pBR-GSS-RSS consisted of *rfp* and *bfp* genes under the regulation of the green light-inducible promoter P_*cpcG2*_ [7] and constitutive strong P_*trcΔLacO*_ promoter [26], which were modified by adding a G-box sequence between the P_*trcΔLacO*_ and the reporter gene, *bfp*. The expression of *rfp* was induced by P_*cpcG2*_ under green light illumination and that of *bfp* was induced by the P_*trcΔLacO*_ promoter, modified by the addition of a G-box sequence, under red light illumination. Moreover, these effects were successfully observed using only a single photosensor.

The transcriptional level induced by *cpcG2* promoter was seemed to be slower than the one in *trcΔLacO* promoter (Fig. 2a, c). By defining the normalized arbitrary unit (nAU), which is the proportion of AU at certain time compared with the maximum AU observed in the experiment (after 18 h of light induction), the speed of the transcription (dnAU/dt), is compared. The speed of the transcription at t  =  6 for *cpcG2* promoter was 4.95 h^−1^, whereas the one for the *trc∆LacO* promoter was 5.29 h^−1^. These results confirm that the transcriptional level is slower in the *cpcG2* promoter, compared with the one in the *trc∆LacO* promoter. This difference would be attributed to the differences of the (− 10) and (− 35) elements between *cpcG2* promoter and *trc∆LacO* promoter. The (− 10) and (− 35) elements of *trc∆LacO* promoter harbors the *E.coli* derived (− 10) and (− 35) elements (5′-TATAAT-3′ and 5′-TTGACA-3′), which is the Type I promoter, typically recognized by sigma factor, σ^70^, of *E. coli*. In contrary, *cpcG2* promoter (5′-ATTCAA-3′ and 5′-AACCGA-3′) is derived from cyanobacteria PCC6803, and lack σ^70^ promoter consensus sequence, therefore, the transcription of this exogenous promoter was slower than the one from *trc∆LacO* promoter [26, 27].

During cultivation under pseudo-continuous cultivation, the transcription level after 30 h decreased. The decrease in transcription might be attributed to the change of the status of the cells due to the low dilution rate we employed. The dilution rate achieved in this pseudo-continuous cultivation was 0.021 h^−1^, which was much lower than the maximum specific growth rate of *E.coli* observed in this study, which is 1.9 h^−1^ where the highest transcription would be kept. Under continuous cultivation, the specific growth rate will be synchronized with the dilution rate, therefore, under this pseudo-continuous cultivation, the status of the cells would be later log phase or stational phase, where the cellular activity including transcriptional activity gradually decreases.

The multichromatic control of a gene expression system in *E. coli* has been reported previously. The expression of the *lacZ* gene was regulated by utilizing two photosensors, an engineered *Cph8* chimera and a cyanobacterial CcaS photoreceptor, and their respective response regulators, OmpR and CcaR [28, 29]. Although our current achievements were not directly compared with these-multi-sensor-based dual light-regulated gene expression systems, the utilization of a single photosensor, the CcaS/CcaR green light-sensing system derived from PCC6803 for the dual control of a light-regulated gene expression system, offers the benefit of more simpler plasmid circuit design, which will affect in the transcription factor involved as well as the regulation system, and be a significant contribution of this system.

The technology used in this study was designed for the application in the regulation of biosynthetic pathways that require multiplex control at different steps using different light colors. The results of this study hold great promise for application in cyanobacterial bioprocesses. The utilization of phytochrome-based family photoreceptors, will provide full control, and various light sensors connected to different transcription factors are available, which allows the multichromatic control of several transcription units using light [30–32]. Moreover, a light-based regulation system is beneficial, as neither additional expensive chemicals nor physical inducers are needed; thus, this system can be easily applied. A regulatory system that can be switched on and off by shifting two different light colors will facilitate a gene expression system that can be tightly regulated at specific times and locations.

## Conclusions

In this study, an artificial CCA system was successfully developed to realize a multiple gene expression system, which was regulated by two colors, red and green lights, using a single photosensor, the CcaS/CcaR system derived from *Synechocystis* sp. PCC6803. The designed and constructed artificial CCA gene expression system regulated the expression of two genes, *rfp*, and *bfp*, under the red and green light; *bfp* expression was induced under red light while *rfp* expression was repressed, and *rfp* expression was induced under green light while *bfp* expression was repressed, as we expected. Moreover, the artificial CCA system functioned repeatedly during the red and green light exposure cycles; the expression of BFP increased under red light exposure while RFP was repressed, and that of RFP increased under green light exposure while that of BFP was repressed. These results demonstrate the potential application of this gene expression system for production of multiple metabolites in a varieties of microorganisms, including cyanobacteria.

## Methods

### Construction of plasmid encoding red light-regulated gene expression system

**Fig. 4:**
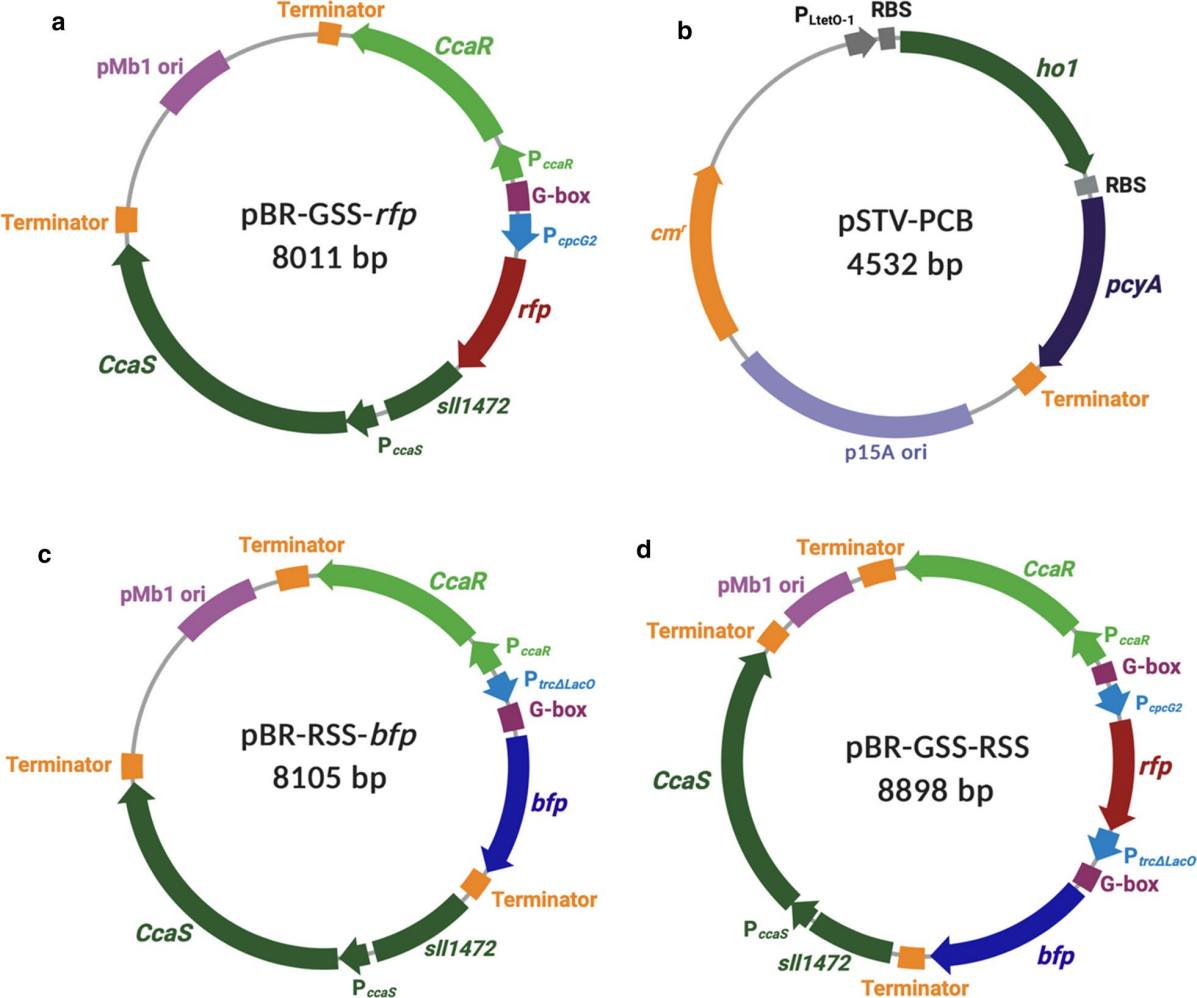
Plasmid used in this research. **a** pBR-GSS-*rfp*, encodes for *rfp* gene driven by P_*cpcG2*_ [12]. **b** pSTV-PCB, encodes protein PCB production [11]. **c** pBR-RSS-*bfp*, encodes *bfp* gene driven by P_*trcΔLacO*_ with an additional G-box upstream of the reporter gene, *bfp*. **d** pBR-GSS-RSS, encodes for *rfp* and *bfp* gene driven by P_*cpcG2*_ and P_*trcΔLacO*_, respectively, with an additional G-box upstream of the reporter gene, *bfp*

The gene encoding the blue fluorescent protein, *bfp* (BBa_K592024) followed by a terminator (BBa_B1006) derived from the BioBrick collection [24], was inserted downstream of the constitutive P_*trcΔLacO*_ promoter, by overlap PCR. The P_*trcΔLacO*_ promoter was modified by inserting a G-box sequence (CTTTCCGATTTCTTTACGATTT), upstream of the reporter gene, *bfp*. The PCR products were assembled corresponding to the *Bam*HI and *Xba*I sites of pBR-GSS-*rfp* (Fig. 4a). pBR-GSS-*rfp* contains the CcaS/CcaR two-component green light-regulated system [11], and was digested with a similar restriction enzyme to omit the *rfp* gene under the regulation of the P_*cpcG2*_ promoter from the plasmid construction. The resulting plasmid was designated pBR-RSS-*bfp* (Fig. 4c), which was used as a control plasmid for red light illumination. Cloning was performed using *E. coli* DH5α as the host, and the pSTV-PCB plasmid [11] which encodes protein PCB production (Fig. 4b).

### Construction of plasmid encoding dual control of light-regulated gene expression system

A designated plasmid harboring dual control of the light-regulated system was constructed by inserting the P_*trcΔLacO*_ promoter, which has been modified as previously described in the Methods section, followed by the *bfp* gene encoding blue fluorescent protein (BBa_K592024) and a terminator (BBa_B1006), derived from BioBrick collection [24], downstream of the *rfp* gene corresponding to the *Bam*HI and *Xba*I sites of pBR-GSS-*rfp* [12], carrying the CcaS/CcaR two-component regulation system from *Synechocystis* sp. PCC6803 (PCC6803) by overlap PCR. The final designated plasmid, pBR-GSS-RSS (Fig. 4d) was cloned using *E. coli* DH5α as the host and the pSTV-PCB plasmid [11], encoding protein PCB production. All constructed plasmids are shown in Fig. 4, and the components are described in Table 1.

**Table 1:** Plasmid construction use in this study

Plasmid name	Feature	Sources
pBR-GSS-*rfp*	Plasmid encodes *ccaS*, *ccaR*, and *rfp* genes transcriptionally driven by P_*cpcG2*_ promoter under green light	[12]
pSTV-PCB	Plasmid encodes protein phycocyanobilin (PCB) production	[11]
pBR-RSS-*bfp*	Plasmid encodes *ccaS*, *ccaR,* and *bfp* genes transcriptionally driven by P_*trcΔLacO*_ promoter under red light	In this study
pBR-GSS-RSS	Plasmid encodes assembly of pBR-GSS-*rfp* and pBR-RSS-*bfp*, transcriptionally driven by P_*cpcG2*_ and P_*trcΔLacO*_ under green and red light, respectively	In this study

### Evaluation of plasmid encoding for red light-regulated gene expression system

Transformants of *E. coli* harboring the plasmid encoding pBR-RSS-*bfp* were pre-cultured in tubes containing 2 mL of fresh LB broth medium with appropriate antibiotics (100 μg/mL ampicillin and 30 μg/mL chloramphenicol), at 37 °C, in an incubator shaker at 140 rpm, overnight in the dark. The cell culture was transferred to 20 mL of fresh LB broth medium with appropriate antibiotics, as described above, in 100-mL Erlenmeyer flasks with the initial optical density at 595 nm (OD _595 nm_  =  0.02) and incubated at 30 °C, in an incubator shaker at 110 rpm, under dark conditions until they reached the exponential phase, which was confirmed at OD 595 nm. After cell growth reached the exponential phase, half of the culture was exposed to red light at an intensity of 40 μmol photon m^−2^ s^−1^, measured using a light meter (LI-250A, LI-COR, Bioscience) and the other half maintained under green light (40 μmol photon m^−2^ s^−1^). During cultivation, samples were withdrawn from the culture to evaluate the effect of the different colors at the transcriptional and translational levels.

### Evaluation of CCA under repeated red and green light illumination

To investigate whether the CCA system functioned after repeated red and green light illumination, the effects of light on the constructed plasmid pBR-GSS-RSS was evaluated under pseudo-continuous conditions according to [25] with some modifications for *E. coli*. In a 500-mL Erlenmeyer flask, containing 150 mL of fresh LB medium with appropriate antibiotics (100 μg/mL ampicillin and 30 μg/mL chloramphenicol), transformants were cultivated at 30 °C, in an incubator shaker at 140 rpm, under the dark conditions until cell growth reached the exponential phase. After the transformants reached the exponential phase, cells were exposed to continuous red, continuous green, or repeated red and green (6 h of red light followed by 6 h of green light) light illumination. Every 12 h, 37.5 mL of culture was replaced with fresh medium containing appropriate antibiotics (100 μg/mL ampicillin and 30 μg/mL chloramphenicol). During cultivation, samples were withdrawn from the culture every 3 h to evaluate the effects of each condition on each designated reporter gene at the transcriptional and translational levels (Additional file 1: Figure S2).

### Transcriptional analysis of *rfp* and *bfp* reporter gene by quantitative PCR (qPCR)

During cultivation, 1 mL of *E. coli* cells harboring pBR-RSS-*bfp* or pBR-GSS-RSS was periodically collected for RNA extraction. RNA was extracted by centrifugation at 4000×*g* for 10 min at 25 °C, using a TRI reagent base of chloroform phenol (Molecular Research Inc.). The isolated RNA was treated with DNase to remove genomic DNA and reverse transcribed into cDNA using a PrimeScript^®^ RT reagent kit with gDNA Eraser (Takara Bio Inc.). qPCR was performed to measure the expression of *rfp* and *bfp* reporter genes and the 16S ribosomal RNA *(rRNA)* housekeeping genes of *E.coli* using SYBR^®^ Premix Ex TaqTM II (Tli RNase H Plus, Takara Bio Inc.). The expression of these genes at the transcriptional level was analyzed using the ∆∆Ct method and normalized by calculating the expression of the 16S *rRNA* housekeeping gene.

### Evaluation of red and green light-regulated protein expression

To monitor the RFP and BFP protein expression levels, 700 μL of each culture was periodically harvested and centrifuged at 12,000×*g* for 2 min at 25 °C, and the supernatant was discarded. Each cell pellet was resuspended in 200 μL of phosphate-buffered saline (PBS); this procedure was repeated twice. The resuspended cell pellet was transferred to a black 96-well plate. The fluorescence of each protein was measured using a plate reader (Variouskan flash spectral scanning microplate reader, Thermo Scientific) with excitation and emission wavelengths (RFP at 584 nm and 607 nm; BFP 399 nm and 456 nm, respectively). The cell density at OD 595 nm was measured after transferring 20 μL from each cell suspension to a clear 96-well plate and diluting with 180 μL of PBS.

## Supplementary Information

**Additional file 1: Figure S1.** Scheme of CCA system with red and green light-regulated gene expression system, derived from *Fremyella diplosiphon* [14] with some modifications. a Under red light, RcaE, RcaF, and RcaC are phosphorylated. Phosphorylated RcaC binding to L-box within the promoter region activates *pcyA* and *cpc2* transcription and represses *cpeC* transcription. b Under green light, RcaE, RcaF, and RcaC are dephosphorylated. Unphosphorylated RcaC does not bind to L-box; consequently, *pcyA* and *cpc2* transcription is deactivated and *cpeC* is transcribed. **Figure S2.** Experimental scheme of *E. coli* transformants harboring pBR-RSS-*bfp* and pBR-GSS-RSS under pseudo-continuous cultivation [25].

## Data Availability

Not applicable.
