# Supplementary material for: Artificial complementary chromatic acclimation gene expression system in Escherichia coli
Source: Microb Cell Fact. 2021 Jul 5;20:128. doi: 10.1186/s12934-021-01621-3 (PMC8256508; doi:10.1186/s12934-021-01621-3)
Supplement: Supplementary file 1 — Additional file 1: Figure S1. Scheme of CCA system with red and green light-regulated gene expression system, derived from Fremyella diplosiphon [14] with some modifications. a Under red light, RcaE, RcaF, and RcaC are phosphorylated. Phosphorylated RcaC binding to L-box within the promoter region activates pcyA and cpc2 transcription and represses cpeC transcription. b Under green light, RcaE, RcaF, and RcaC are dephosphorylated. Unphosphorylated RcaC does not bind to L-box; consequently, pcyA and cpc2 transcription is deactivated and cpeC is transcribed. Figure S2. Experimental scheme of E. coli transformants harboring pBR-RSS-bfp and pBR-GSS-RSS under pseudo-continuous cultivation [25]. [file 12934_2021_1621_MOESM1_ESM.pdf]

Artificial complementary chromatic acclimation gene expression system in *Escherichia coli*

Dwi Ariyanti[1, 2], Kazunori Ikebukuro[1*], Koji Sode[3*]

***Correspondences:** ikebu@cc.tuat.ac.jp; ksode@email.unc.edu

[1]Department of Biotechnology and Life Science, Graduate School of Engineering, Tokyo University of Agriculture and Technology, 2-24-16 Naka-cho, Koganei, Tokyo 184-8588, Japan

[2]Faculty of Biotechnology, Sumbawa University of Technology, Olat Maras, Moyo Hulu, Sumbawa, West Nusa Tenggara 84371, Indonesia

[3]Joint Department of Biomedical Engineering, The University of North Carolina at Chapel Hill and North Carolina State University, Chapel Hill, NC 27599, United States

Supplementary information

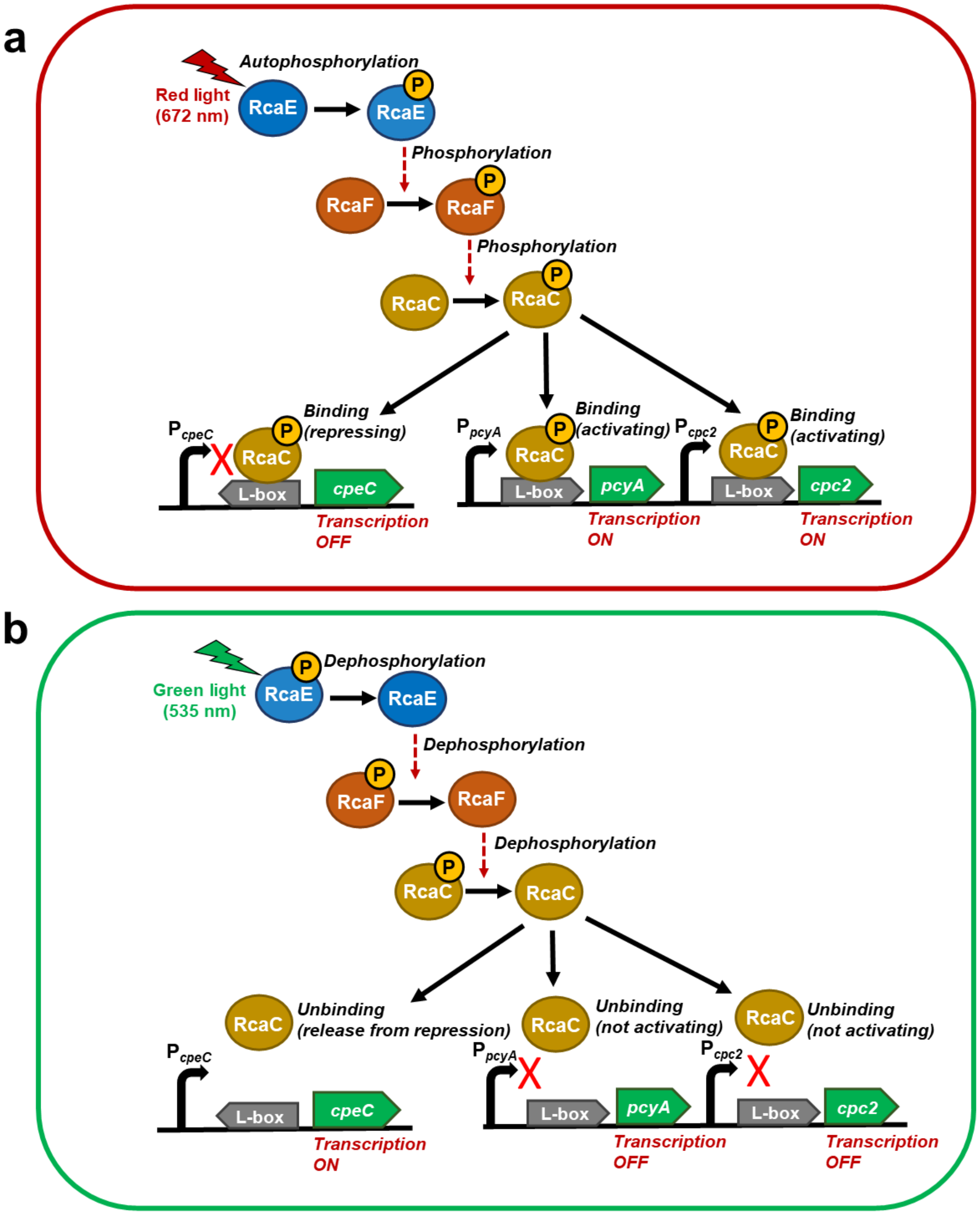

Figure S1. Scheme of CCA system with red and green light-regulated gene expression system, derived from *Fremyella diplosiphon* [14] with some modifications. **a** Under red light, RcaE, RcaF, and RcaC are phosphorylated. Phosphorylated RcaC binding to L-box within the promoter region activates *pcyA* and *cpc2* transcription and represses *cpeC* transcription. **b** Under green light, RcaE, RcaF, and RcaC are dephosphorylated. Unphosphorylated RcaC does not bind to L-box; consequently, *pcyA* and *cpc2* transcription is deactivated and *cpeC* is transcribed.

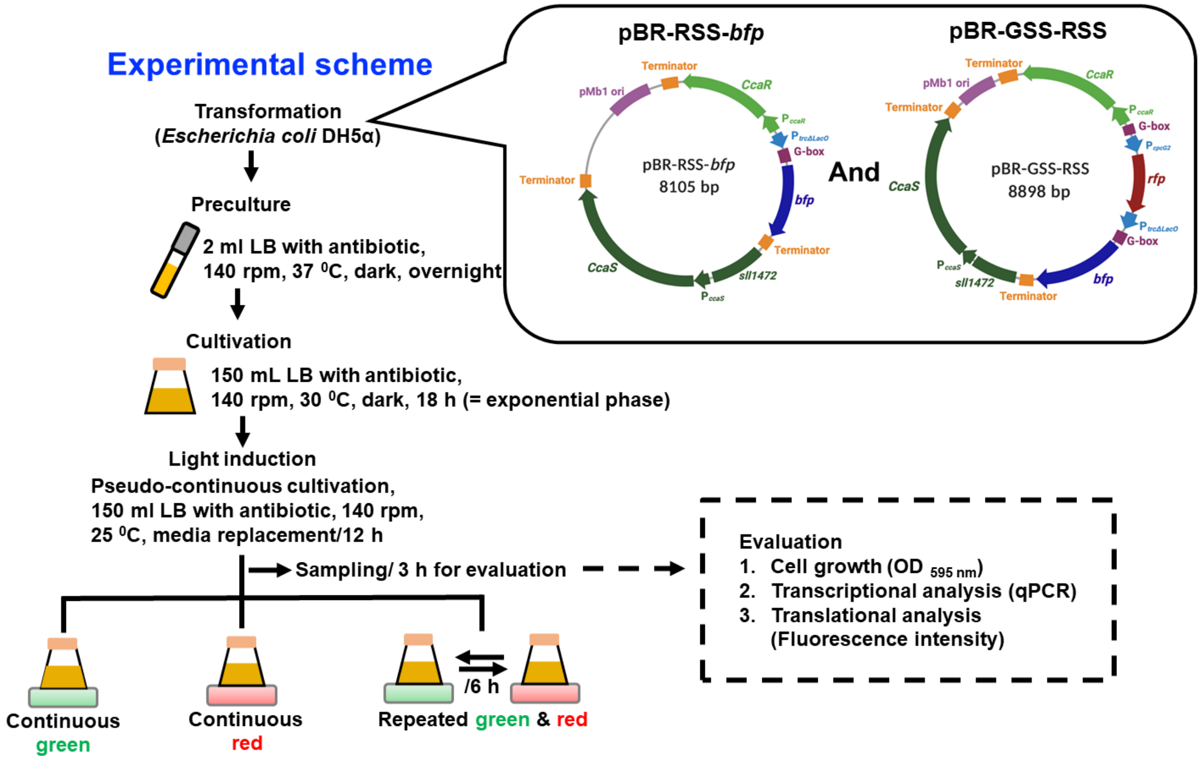

Figure S2. Experimental scheme of *E. coli* transformants harboring pBR- RSS-*bfp* and pBR-GSS-RSS under pseudo-continuous cultivation [25].
